# Neutral ceramidase‐enriched exosomes prevent palmitic acid‐induced insulin resistance in H4IIEC3 hepatocytes

**DOI:** 10.1002/2211-5463.12125

**Published:** 2016-10-20

**Authors:** Qun Zhu, Rongping Zhu, Junfei Jin

**Affiliations:** ^1^ Department of Endocrinologythe Second Affiliated Hospital of Nanjing Medical UniversityJiangsuChina; ^2^ China‐USA Lipids in Health and Disease Research CenterGuilin Medical UniversityGuangxiChina; ^3^ Laboratory of Hepatobiliary and Pancreatic SurgeryAffiliated Hospital of Guilin Medical UniversityGuangxiChina; ^4^ Guangxi Key Laboratory of Molecular Medicine in Liver Injury and RepairGuilin Medical UniversityGuangxiChina

**Keywords:** ceramide, exosome, insulin resistance, neutral ceramidase, palmitic acid, reactive oxygen species

## Abstract

Oversupply of free fatty acids such as palmitic acid (PA) from the portal vein may cause liver insulin resistance. Production of reactive oxygen species plays a pivotal role in PA‐induced insulin resistance in H4IIEC3 hepatocytes. Recently, we found that exosomes secreted from INS‐1 cells that were transfected with neutral ceramidase (NCDase) plasmids had raised NCDase activity; these NCDase‐enriched exosomes could inhibit PA‐induced INS‐1 cell apoptosis. Here, we showed that PA reduced insulin‐stimulated tyrosine phosphorylation of insulin receptor substrate 2 and decreased insulin‐stimulated uptake of the fluorescent glucose analog 2‐NBDG, confirming that insulin resistance occurred in PA‐treated H4IIEC3 cells. Moreover, NCDase‐enriched exosomes from INS‐1 cells rescued PA‐induced H4IIEC3 insulin resistance and blocked PA‐induced reactive oxygen species production in which ceramide was involved.

**Abbreviations**
2‐NBDG: 2‐[*N*‐(7‐nitrobenz‐2‐oxa‐1,3‐diazol‐4‐yl) amino]‐2‐deoxy‐d‐glucose
FFA: free fatty acid
GSK‐3: glycogen synthase kinase 3
H2DCFDA: 2,7‐dichlorofluorescin diacetate
IRS1: insulin receptor substrate 1
IRS2: insulin receptor substrate 2
NCDase‐Exo: NCDase‐enriched exosome
NCDase: neutral ceramidase
PA: palmitic acid
PI3K: phosphoinositide 3‐kinase
ROS: reactive oxygen species
S1P: sphingosine‐1‐phosphate

Impaired hepatic insulin sensitivity leads to more glucose production in the liver, resulting in hyperglycemia 1 and development of type 2 diabetes 2. Too much dietary lipid, especially free fatty acids (FFAs), passing through the portal vein and being deposited in the liver might cause the liver's insulin resistance 2; an increase in FFAs in blood decreased insulin sensitivity in some target tissues 3. A report in mice confirmed that oxidative stress induced by lipid caused insulin resistance in liver 4, and reactive oxygen species (ROS) production plays a pivotal role in palmitic acid (PA)‐induced insulin resistance in rat H4IIEC3 hepatocytes 2.

In the sphingolipid metabolic pathway, neutral ceramidase (NCDase) is one of the most important enzymes; the catalytic action of NCDase leads to ceramide hydrolysis and sphingosine generation. It is well known that ceramide is a key molecule in the sphingolipid pathway, and is involved in many physiological and pathological processes such as apoptosis, differentiation and insulin resistance. Therefore, as an important ceramide hydrolyzing enzyme, NCDase *per se* is also involved in the aforementioned processes. It has been reported that exosomes function to transport a variety of substances 5, and can be internalized into cells by membrane fusion or endocytosis 6, as a result of which their contents would be transferred to the recipient cells. Our previous study confirmed that NCDase‐containing exosomes isolated from the culture medium from INS‐1 cells exposed to a low concentration of cytokines inhibited apoptosis induced by a high concentration of cytokines 7. Very recently, we found that exosome secreted from INS‐1 cells transfected with NCDase plasmids had raised NCDase activity, and these NCDase‐enriched exosomes (NCDase‐Exos) could inhibit PA‐induced INS‐1 cell apoptosis (S. Tang, F. Luo, Y. Feng, X. Wei, H. Miao, Y. Lu, Y. Tang, D. Ding, J. Jin and Q. Zhu, unpublished data). The action of NCDase‐Exos on PA‐induced insulin resistance is investigated in the present study. We hypothesized that NCDase‐Exos from INS‐1 cells could prevent PA‐induced H4IIEC3 insulin resistance by decreasing ROS production.

## Materials and methods

### Cells and materials

All cells were incubated in a humidified atmosphere at 37 °C containing 5% CO_2_.

Rat INS‐1 cells were cultured in RPMI 1640 medium supplemented with 10% heat‐inactivated fetal bovine serum, 50 μm 2‐mercaptoethanol, 2 mm l‐glutamine, 10 mm HEPES, 1 mm sodium pyruvate, 100 units·mL^−1^ penicillin, and 0.1 mg·mL^−1^ streptomycin. Stable INS‐1 cells transfected with pEGFP‐C3‐NCDase (a gift from Y. Hannun, Medical University of South Caronia, Charleston, SC, USA) or control vector pEGFP‐C3 (Clontech Laboratories, Inc., Mountain View, CA, USA) were established in our recent study (S. Tang, F. Luo, Y. Feng, X. Wei, H. Miao, Y. Lu, Y. Tang, D. Ding, J. Jin and Q. Zhu, unpublished data), as in a previous study 8. H4IIEC3 rat hepatoma cells were cultured in Dulbecco's modified Eagle's medium (DMEM) supplemented with 10% fetal bovine serum, 100 units·mL^−1^ penicillin and 0.1 mg·mL^−1^ streptomycin, and 80–90% confluent cells were used in all experiments 2.

Antibody against total insulin receptor substrate 2 (t‐IRS2) was obtained from Upstate Biotechnology Inc. (Lake Placid, NY, USA); antibody against the phosphorylated IRS2 (p‐IRS2) was from Cell Signaling Technology (Danvers, MA, USA). Polyvinylidene difluoride (PVDF) membranes were from Millipore (Billerica, MA, USA). ECL Plus western blotting detection reagents were from GE Healthcare (Piscataway, NJ, USA). Porcine pancreas insulin and sodium palmitate were obtained from Sigma‐Aldrich (St Louis, MO, USA). 2,7‐Dichlorofluorescin diacetate (H_2_DCFDA) was purchased from Wako (Osaka, Japan). 2‐[*N*‐(7‐Nitrobenz‐2‐oxa‐1,3‐diazol‐4‐yl) amino]‐2‐deoxy‐d‐glucose (2‐NBDG) was from Invitrogen (USA). The PI3K ELISA kit was from Echelon Biosciences (Salt Lake City, UT, USA). Both the Pierce AKT colorimetric in‐cell ELISA kit and the Pierce GSK3 α/β colorimetric in‐cell ELISA kit were purchased from Thermo Scientific (Rockford, IL, USA). Mouse/Human/Rat Phospho‐IRS1 (Ser307) ELISA Kit was from LifeSpan BioSciences (Seattle, WA, USA).

### Preparation of exosomes and treatment

The conditioned medium from INS‐1 cells transfected with pEGFP‐C3‐NCDase or the control vector pEGFP‐C3 was prepared and then used for exosome isolation as in our previous report 7. In brief, after conditioned medium was ultracentrifugated at 110 000 ***g*** and 4 °C for 70 min, the pellets were resuspended in 1× PBS for a second ultracentrifugation. The protein concentration in exosomes was measured and 10 μg of exosomes was added to H4IIEC3 hepatocytes seeded in six‐well plates.

### NCDase activity assay

The enzymatic activity of NCDase in exosome was determined by measuring the amount of sphingosine generated from ceramide as in our previous report 9.

### Western blot

H4IIEC3 hepatocytes with confluence of 80–90% were treated with PA for 16 h. After 15 min stimulation by insulin at a concentration of 1 ng·mL^−1^, the cells were collected and lysed. After centrifugation, 10 μg of sample from the supernatant was loaded for detection of t‐IRS2. For detection of p‐IRS2, 400 μg of protein from the supernatant was immunoprecipitated at 4 °C for 2 h with the antibody against p‐IRS2 and protein G beads before loading. The membrane was incubated with primary antibody, then with second antibody, and finally detected and exposed to X‐ray film, as described previously 2.

### 2‐NBDG uptake

2‐NBDG, a fluorescent d‐glucose analog, was used to determine glucose uptake by H4IIEC3 hepatocytes. Cells were treated with PA for 16 h, then stimulated with 100 nm insulin for 15 min, and finally incubated with 50 mm 2‐NDBG for 20 min. After that, cells were washed with 1× PBS three times and fluorescence densities were measured (excitation wavelength: 485 nm; emission wavelength: 535 nm) and normalized per milligram of total protein. The nonspecific uptake of 2‐NBDG was excluded as in a previous report 10.

### ROS production

H2DCFDA, a fluorescent probe, was used to determine ROS production in H4IIEC3 hepatocytes according to a previous report 2. H4IIEC3 hepatocytes with a 70–80% confluence were exposed to PA for 16 h, then treated with 10 μm H_2_DCFDA for 30 min and the fluorescence was measured.

### PA preparation and final concentration

Palmitic acid was prepared as in our previous report 11 and was free of bovine serum albumin. It was dissolved at 70 °C in a solution of 0.1 m NaOH and 70% ethanol to produce PA stock solution (50 mm); the final concentration of PA was 0.25 mm.

### Statistical analysis

The data from three independent experiments are expressed as the mean ± SD and were analyzed by Student's *t* test or ANOVA. A value of *P* < 0.05 was designated as statistically significant.

## Results

**Figure 1:**
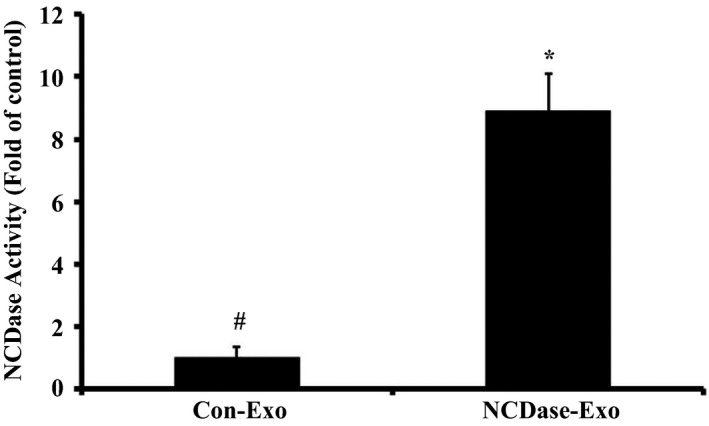
Neutral ceramidase‐Exos have a higher NCDase activity than Con‐Exos. Exosomes from INS‐1 cells transfected with pEGFP‐C3‐NCDase (NCDase‐Exo) had higher NCDase activity compared with the exosomes from INS‐1 cells transfected with the control vector pEGFP‐C3 (Con‐Exo), # vs. *: *P <* 0.05. NCDase activity in NCDase‐Exos and Con‐Exos was determined by HPLC and data from triplicate experiments are expressed as fold of control (mean ± SD).

Very recently, we found that exosomes secreted from INS‐1 cells that were transfected with NCDase plasmids had raised NCDase activity, and these NCDase‐enriched exosomes could inhibit PA‐induced INS‐1 cell apoptosis. Further work confirmed that a change in the ceramide/sphingosine‐1‐phosphate (S1P) ratio might be the mechanism (S. Tang, F. Luo, Y. Feng, X. Wei, H. Miao, Y. Lu, Y. Tang, D. Ding, J. Jin and Q. Zhu, unpublished data). Therefore, we wanted to know whether or not these NCDase‐enriched exosomes could prevent PA‐induced H4IIEC3 insulin resistance. Firstly, INS‐1 cells were transfected with pEGFP‐C3‐NCDase; the exosomes isolated from the culture medium of these cells are termed NCDase‐Exos. Similarly, exosomes from INS‐1 cells transfected with the control vector pEGFP‐C3 are termed Con‐Exos. As shown in Fig. 1, the NCDase‐Exos had higher NCDase activity compared with the Con‐Exos.

### NCDase‐Exos could rescue PA‐induced insulin resistance

**Figure 2:**
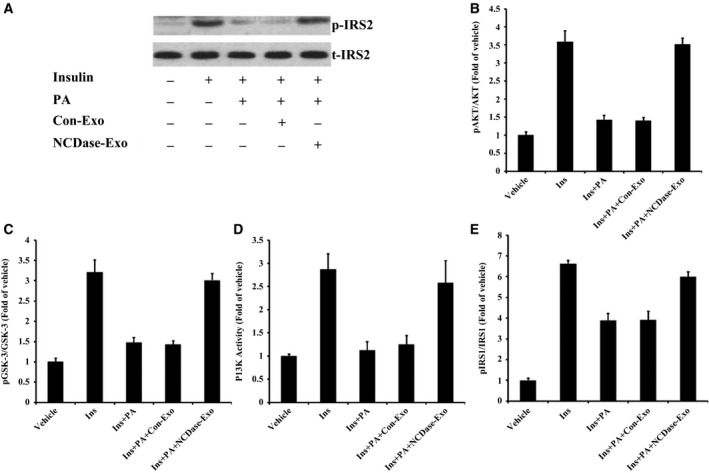
Neutral ceramidase‐Exos can rescue the effect of PA on insulin‐stimulated phosphorylation of IRS‐2 or other related proteins in H4IIEC3 cells. H4IIEC3 hepatocytes were treated with PA alone or along with exosomes for 16 h, and then incubated with 1 ng·mL^−1^ of insulin for 15 min. After that, cells were collected and lysed for western blotting with t‐IRS2 antibody. To examine p‐IRS2 (A) total proteins from H4IIEC3 cells were firstly immunoprecipitated with p‐IRS2 antibody and then western blotting was performed. Cell lysates from H4IIEC3 hepatocytes were analyzed for phosphorylation or activity of AKT (B) GSK‐3 (C) PI3K (D) and IRS‐1 (E) by ELISA. Vehicle vs. Ins (insulin): *P <* 0.05; Ins vs. Ins+PA:* P <* 0.05; Ins+PA vs. Ins+PA+Con‐Exo: *P >* 0.05; Ins+PA+Con‐Exo vs. Ins+PA+NCDase‐Exo: *P <* 0.05.

Consistent with previous findings 2, PA reduced insulin‐stimulated tyrosine phosphorylation of insulin receptor substrate 2 (IRS2) in H4IIEC3 hepatocytes (Fig. 2A); however, NCDase‐Exos, but not Con‐Exos, could partly rescue the effect of PA on insulin‐stimulated tyrosine phosphorylation of IRS2 (Fig. 2A). Similar to this result, PA also decreased insulin‐dependent activation of AKT (Fig. 2B), glycogen synthase kinase 3 (GSK‐3; Fig. 2C), phosphoinositide 3‐kinase (PI3K; Fig. 2D) and insulin receptor substrate 1 (IRS1; Fig. 2E) in hepatocytes, and NCDase‐Exos could reverse these effects.

**Figure 3:**
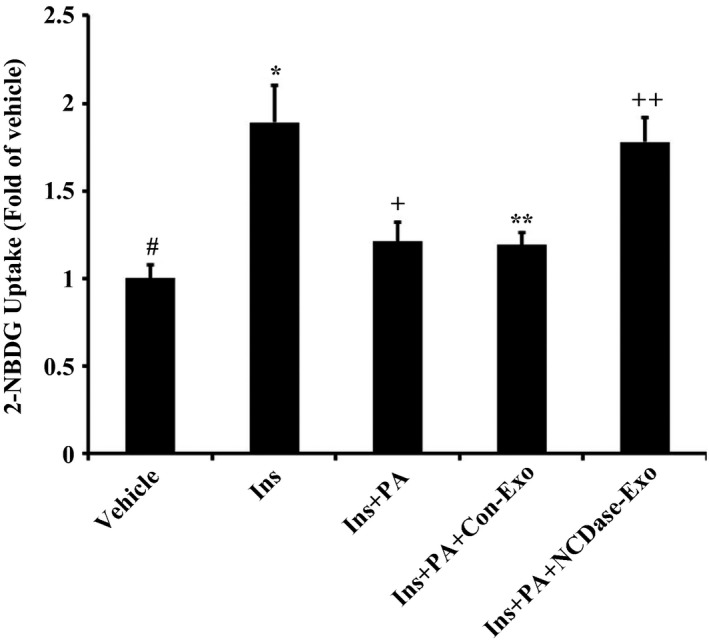
Effect of NCDase‐Exos on insulin‐stimulated 2‐NBDG uptake in PA‐treated H4IIEC3 cells. H4IIEC3 hepatocytes were treated with PA alone or along with exosomes for 16 h, then stimulated with 100 nm insulin for 15 min, and finally incubated with 50 mm 2‐NDBG for 20 min, and fluorescence densities were measured as described in Methods. Data from three determinations are normalized by milligram of total protein and are expressed as fold of control (means ± SD). # vs. *: *P <* 0.05; * vs. +: *P <* 0.05; * vs. **: *P <* 0.05; + vs. **: *P >* 0.05; ** vs. ++: *P <* 0.05.

In addition, PA decreased insulin‐stimulated 2‐NBDG uptake in H4IIEC3 hepatocytes (Fig. 3), and NCDase‐Exos, but not Con‐Exos, could inhibit the effect of PA on insulin‐stimulated 2‐NBDG uptake (Fig. 3). These data revealed that NCDase‐Exos could rescue PA‐induced insulin resistance.

### NCDase‐Exos could reduce PA‐induced ROS production

**Figure 4:**
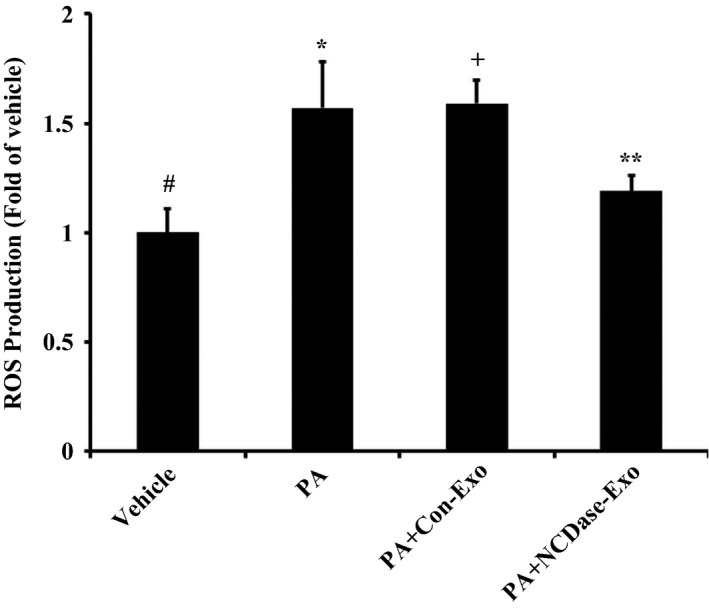
Effect of NCDase‐Exos on ROS production induced by PA in H4IIEC3 cells. H4IIEC3 hepatocytes with a 70–80% confluence were exposed to PA alone or PA along with exosomes for 16 h, then treated with 10 μm H_2_DCFDA for 30 min and the fluorescence measured. Data from three determinations are expressed as fold of control (means ± SD). # vs. *: *P <* 0.05; # vs. +: *P <* 0.05; * vs. +: *P >* 0.05; + vs. **: *P <* 0.05.

Because reactive oxygen species (ROS) were a cause of palmitate‐induced insulin resistance in H4IIEC3 hepatocytes 2, we wanted to investigate the effect of NCDase‐Exos on ROS production in H4IIEC3 hepatocytes exposed to PA. As expected, PA led to ROS production in H4IIEC3 hepatocytes (Fig. 4), and NCDase‐Exos could partially block PA‐induced ROS production.

### Ceramide plays a key role in the effect of NCDase‐Exos on the reduction of PA‐mediated ROS production

**Figure 5:**
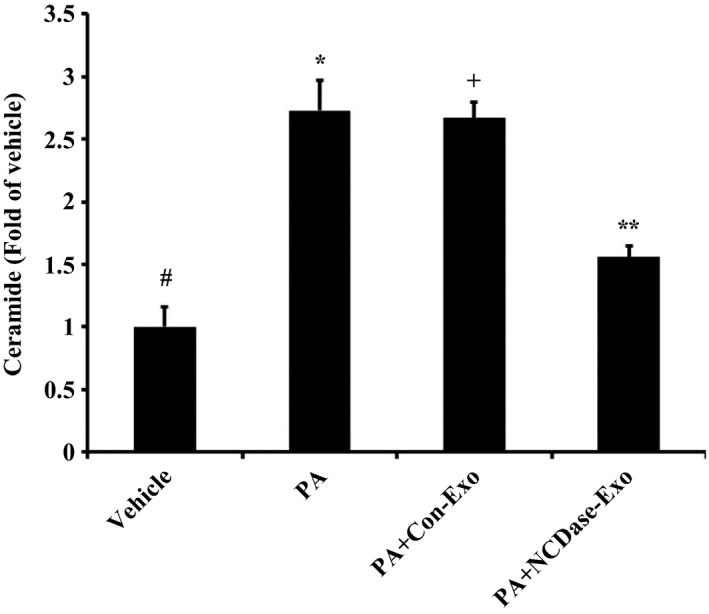
Effect of NCDase‐Exos on ceramide elevation induced by PA in H4IIEC3 cells. H4IIEC3 hepatocytes with a 70–80% confluence exposed to PA alone or PA along with exosomes for 16 h were collected and ceramide in these cells was measured as in our previous report 25. Data from three determinations are expressed as fold of control (means ± SD). # vs. *: *P <* 0.05; # vs. +: *P <* 0.05; * vs. +: *P >* 0.05; + vs. **: *P <* 0.05.

**Figure 6:**
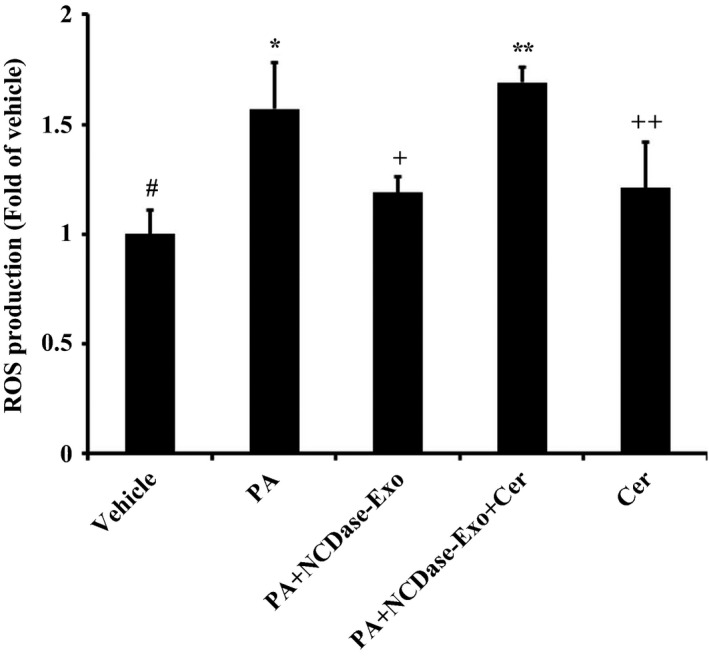
Exogenous C6‐ceramide could ameliorate the effect of NCDase‐Exos on ROS production of H4IIEC3 hepatocytes under PA treatment. H4IIEC3 hepatocytes with a 70–80% confluence were exposed to PA alone or PA along with exosomes for 10 h, then 40 μm C6‐ceramide (Cer) was added and cells were incubated for a further 6 h; finally, H4IIEC3 hepatocytes were treated with 10 μm H_2_ DCFDA for 30 min and the fluorescence was measured. Data from three determinations are expressed as fold of control (means ± SD). # vs. *: *P <* 0.05; * vs. +: *P <* 0.05; + vs. **: *P <* 0.05; # vs. ++: *P <* 0.05.

Because ceramide is a cause of ROS production 12 and is involved in palmitate‐induced insulin resistance 13, we investigated the role of ceramide in the effect of NCDase‐Exos on ROS reduction in H4IIEC3 hepatocytes exposed to PA. As expected, PA treatment led to a ceramide increase in H4IIEC3 hepatocytes (Fig. 5), while PA had no effect on the levels of sphingosine and S1P under our conditions (data not shown), and NCDase‐Exos could partly block this ceramide elevation. Interestingly, adding C6‐ceramide could ameliorate the effect of NCDase‐Exos on ROS production of H4IIEC3 hepatocytes under PA treatment (Fig. 6).

## Discussion

Exosomes, a distinct kind of nanoparticle with a size from 40 to 100 nm, can be internalized into cells 6 and function in transporting different substances 5, including functional RNAs, lipids, proteins (or enzymes) and even viruses. They therefore act in intercellular transmission 14. Moreover, the altered characteristics of exosome in many diseases indicate that exosomes are beneficial in the areas of diagnosis and therapy, so that the idea emerges that exosomes might be used as a vehicle to deliver drugs 15. Thus we hypothesized that exosomes, secretory vesicles, possibly act as carriers to transport enzymes like NCDase to target cells or organs.

NCDase, an important enzyme in the sphingolipid metabolic pathway, is responsible for hydrolyzing ceramide to produce sphingosine. Some researchers, including ourselves, confirmed that NCDase protects cells from ceramide's cytotoxicity induced by some cytokines such as interferon γ, interleukin 1β and tumor necrosis factor α via up‐regulation of NCDase mRNA and protein 9, 16, 17. Lately we found that a low concentration of a cytokine mixture led to the release of exosomes containing NCDase, and these exosomes could reduce a high concentration of cytokine mixture‐induced cell death 7. Higher NCDase activity appeared in exosomes secreted from INS‐1 cells transfected with human NCDase vectors, and these exosomes (NCDase‐Exos) could inhibit palmitic acid‐induced INS‐1 cell death (S. Tang, F. Luo, Y. Feng, X. Wei, H. Miao, Y. Lu, Y. Tang, D. Ding, J. Jin and Q. Zhu, unpublished data), while the control exosomes (Con‐Exos), isolated from the control vector‐transfected INS‐1 cells, could not.

**Figure 7:**
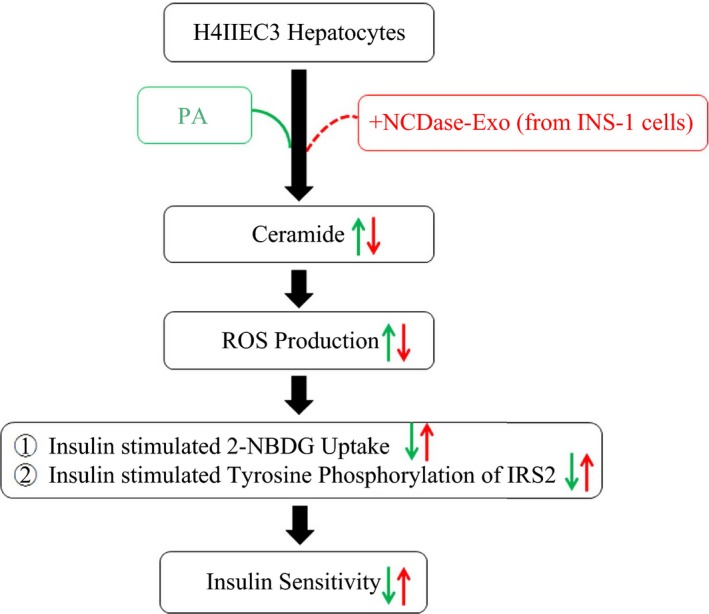
Scheme summarizing the signaling of ceramide–ROS–insulin resistance in PA‐treated H4IIEC3 hepatocytes and the intervention of NCDase‐Exos.

Ceramide is implicated in insulin resistance mediated by FFAs such as PA 18, 19, and ROS generation induced by ceramide was confirmed in many studies 20, 21, 22. An elegant study by Nakamura *et al*. revealed that ROS has a pivotal role in PA‐induced H4IIEC3 hepatocyte insulin resistance 2. The authors could not determine whether ceramide is necessary for ROS production and subsequent insulin resistance, although an increment in the level of intracellular ceramide was observed in H4IIEC3 cells under PA treatment. Therefore, there was a lack of direct evidence about the signaling of ceramide–ROS–insulin resistance in PA‐treated H4IIEC3 cells. In our study, firstly we confirmed in H4IIEC3 cells that PA decreased phosphorylated tyrosine in IRS2 indicating that PA induced insulin resistance, which is in agreement with Nakamura *et al*.'s report 2. Interestingly, the NCDase‐Exos could partially prevent PA‐mediated decrease of insulin sensitivity, which is confirmed by the data from 2‐NBDG uptake stimulated by insulin. NCDase‐Exos could also ameliorate PA‐mediated ROS production and PA‐induced ceramide increase. Exogenous ceramide addition could attenuate the effect of NCDase‐Exos on ROS production in PA‐treated H4IIEC3 hepatocytes, exogenous ceramide alone leading to ROS production in this cell, confirming that ceramide is the upstream signal of ROS production. The findings in this report are summarized in schematic form in Fig. 7. It illustrates the signaling of ceramide–ROS–insulin resistance in PA‐treated H4IIEC3 hepatocytes, and that NCDase‐Exos can inhibit this signaling cascade and rescue PA‐mediated H4IIEC3 insulin resistance.

As we know, ceramide has an important role in the pathogenesis of types 1 and 2 diabetes 23, and ceramide is the key intermediate linking some of the steps to insulin resistance 24. NCDase, a key ceramide‐degrading enzyme, may prove efficacious as a therapeutic to combat insulin resistance, especially as exosome‐packaged NCDase allows easy transportation of NCDase without degradation. A good question is why NCDase in the exosomes is the ‘active’ agent and not some other downstream targets of NCDase action, such as ceramide, sphingosine, or S1P. Because no differences of ceramide, sphingosine, or S1P in NCDase‐Exos and in Con‐Exos were observed (data not shown), these molecules were excluded as the ‘active’ agents in exosomes. As we know, downstream targets of NCDase action include many genes, lipids and metabolites, all of which are also present in exosomes, and therefore the possibility also exists of other targets besides NCDase involvement. However, the limit of this research is that we cannot answer how NCDase‐Exos enter the cells, and how NCDase is released and where it works; further studies are needed to answer these questions.

## Author contributions

QZ conducted experiments, analyzed data and drafted the manuscript. RZ completed the additional experiments, analyzed these data, drafted related figures, and revised the manuscript. JJ designed the experiments, revised the manuscript and obtained grants.
